# A combined strategy of neuropeptide prediction and tandem mass spectrometry identifies evolutionarily conserved ancient neuropeptides in the sea anemone *Nematostella vectensis*

**DOI:** 10.1371/journal.pone.0215185

**Published:** 2019-09-23

**Authors:** Eisuke Hayakawa, Hiroshi Watanabe, Gerben Menschaert, Thomas W. Holstein, Geert Baggerman, Liliane Schoofs

**Affiliations:** 1 Research Group of Functional Genomics and Proteomics, KU Leuven, Leuven, Belgium; 2 Evolutionary Neurobiology Unit, Okinawa Institute of Science & Technology, Okinawa, Japan; 3 Centre for Organismal Studies (COS), Heidelberg University, Heidelberg, Germany; 4 Faculty of Bioscience Engineering, Laboratory for Bioinformatics and Computational Genomics, Ghent University, Ghent, Belgium; 5 CFP/Ceproma, University Antwerpen, Antwerpen, Belgium; 6 VITO, Applied Bio & molecular Systems (ABS), Mol, Belgium; Universite de Rouen, FRANCE

## Abstract

Neuropeptides are a class of bioactive peptides shown to be involved in various physiological processes, including metabolism, development, and reproduction. Although neuropeptide candidates have been predicted from genomic and transcriptomic data, comprehensive characterization of neuropeptide repertoires remains a challenge owing to their small size and variable sequences. *De novo* prediction of neuropeptides from genome or transcriptome data is difficult and usually only efficient for those peptides that have identified orthologs in other animal species. Recent peptidomics technology has enabled systematic structural identification of neuropeptides by using the combination of liquid chromatography and tandem mass spectrometry. However, reliable identification of naturally occurring peptides using a conventional tandem mass spectrometry approach, scanning spectra against a protein database, remains difficult because a large search space must be scanned due to the absence of a cleavage enzyme specification. We developed a pipeline consisting of *in silico* prediction of candidate neuropeptides followed by peptide-spectrum matching. This approach enables highly sensitive and reliable neuropeptide identification, as the search space for peptide-spectrum matching is highly reduced. *Nematostella vectensis* is a basal eumetazoan with one of the most ancient nervous systems. We scanned the *Nematostella* protein database for sequences displaying structural hallmarks typical of eumetazoan neuropeptide precursors, including amino- and carboxyterminal motifs and associated modifications. Peptide-spectrum matching was performed against a dataset of peptides that are cleaved *in silico* from these putative peptide precursors. The dozens of newly identified neuropeptides display structural similarities to bilaterian neuropeptides including tachykinin, myoinhibitory peptide, and neuromedin-U/pyrokinin, suggesting these neuropeptides occurred in the eumetazoan ancestor of all animal species.

## Introduction

Neuropeptides are a highly diverse group of messenger molecules involved in neurotransmission. They are essential for many physiological processes, such as muscle contraction, food digestion, growth, development, and reproduction, as well as more complex behaviours, such as adaptation, learning and memory, and ageing [1]. A neuropeptide is usually encoded in a larger neuropeptide precursor gene, which also encodes an N-terminal signal peptide. The precursor is translated on the rough endoplasmic reticulum, and the signal peptide is removed by a signal peptidase. Afterwards, processing enzymes in the immature secretory granules typically produce one or more mature neuropeptides by processing the protein precursor at cleavage sites. A number of cleavage enzymes, so called prohormone convertases of the furin/subtilisin family with specific recognition patterns, have been identified [2,3]. After cleavage, the processed peptides can undergo various posttranslational modifications (PTMs) [4]. Especially, the N-terminal and/or C-terminal residues are often modified. A frequently observed modification is amidation at the C-terminus of mature neuropeptides, which is usually required for their biological activity. C-terminal amidation involves the enzymatic transformation of a glycine into an alpha-amide by peptidylglycine alpha-amidating monooxygenase [5]. First, peptidylglycine is transformed into peptidyl-alpha-hydroxyglycine in the presence of copper, ascorbate, and molecular oxygen and subsequently converted to peptide alpha-amide and glyoxylate. C-terminal amidation protects the mature peptide from enzymatic degradation by carboxypeptidases. Pyroglutamic acid is a posttranslational modification that is widely observed at N-termini of various neuropeptides and protects the peptide chain from enzymatic degradation by aminopeptidases. In addition to PTMs, many bioactive peptides contain a proline residue in the second or third position from the N-terminus. This feature also protects the peptide from peptidase activity at the N-terminus [6]. All these structural characteristics are widely observed in neuropeptide sequences across the Animal Kingdom [7].

Recent advances in mass spectrometry and liquid chromatography technology have led to the establishment of peptidomics, an efficient technology that combines liquid chromatography with tandem mass spectrometry to identify neuropeptides. Tandem mass spectrometry combined with peptide-spectrum matching tools enables systematic neuropeptide identification [8–10]. Unlike neuropeptide prediction tools that are based on sequence similarities, the peptidomics approach provides evidence for the *in vivo* occurrence of the mature peptides, and also shows the eventual presence of the peptide’s PTMs [11,12]. However, identification of naturally occurring processed peptides by means of conventional peptide-spectrum matching tools remains difficult. Unlike proteomics, in which proteins are identified based on *in vitro* generated enzymatic peptide digests, peptidomics uses naturally occurring peptides already cleaved by processing enzymes. Because cleavage sites in a protein precursor of naturally occurring neuropeptides cannot be predicted with high accuracy [13], peptide-spectrum matching in classical peptidomics technology has to be performed without any enzyme specification. This drawback in peptidomics leads to a huge search space and often results in poor identification confidence values. In addition, all possible PTMs have to be taken into consideration, which further increases the search space for peptide-spectrum matching [14].

A commonly employed approach to identify neuropeptides is based on sequence similarities and has allowed the *in silico* prediction of putative peptide signatures in sequenced genomes and transcriptomes of several animal species [15–18]. Multiple reports have successfully tracked down the evolutionarily conserved neuropeptides within a phylum or between closely related phyla, by means of sequence similarity-based searches against protein databases [19]. This approach is very useful for searching peptide sequences that have been evolutionarily conserved between closely related species. However, when peptides have become evolutionarily diverged, such as evolutionarily ancient organisms, the sequence similarity-based prediction of new neuropeptides may not always be successful. Moreover, within a particular peptide sequence, only a short motif required for the peptide’s biological activity is conserved during evolution [18,20], and the non-peptide-coding region of the precursor is in general not conserved. These issues hamper reliable sequence similarity-based peptide prediction. This is especially true for neuropeptides and corresponding genes, based on homology searches, among evolutionarily distant species, thus necessitating experimental validation. To address these hurdles, we have developed an alternative strategy to identify neuropeptides with high sensitivity and confidence. The strategy involves the construction of a significantly reduced dataset that only comprises mature peptide sequences, cleaved from their predicted protein precursors *in silico*. The latter are extracted from the much larger protein database based on their typical hallmarks (signal peptide, specific cleavage sites, PTMs). Peptide-spectrum matching is performed against the significantly reduced dataset of peptide sequences. The narrowed search space for peptide-spectrum matching enables highly sensitive peptide identification.

Using this approach, we here present a systematic identification of neuropeptides from *Nematostella vectensis*. This sea anemone has an urbilaterian origin and is thus one of the most evolutionarily ancient animals with a nervous system. Urbilaterian origins of neuropeptide signaling systems are still debated. There are four known non-bilaterian metazoan phyla: two phyla that have nervous systems, the Ctenophora (comb jellies) and Cnidaria (e.g., sea anemones and jelly fish); and two phyla that lack nervous systems, the Placozoa (e.g., *Trichoplax*) and the Porifera (sponges). A variety of bioactive neuropeptides have been identified in the cnidarians *Renilla köllikeri* (class Anthozoa) and *Hydra magnipapillata* (class Hydrozoa) [21–27]. The cnidarian genome of *Nematostella* predicts the presence of putative neuropeptide precursors with following C-terminal motifs: RIamides, Rpamides, Rwamides, Lwamides, Itamide, Mtamide, Vramide, Rramide, Pgamides, Rgamides, Pvamides, and LVamide [28,29]. However, none of these neuropeptides appear to be orthologues of bilaterian neuropeptides. This complicates our understanding of the evolution of neuropeptides. To get deeper insight into the puzzle of neuropeptide evolution, identification of neuropeptides in urbilaterian animals is not only fascinating, but of utmost importance. We successfully identified 20 neuropeptides in *Nematostella*, many of which have not been predicted or annotated as neuropeptides before [28].

## Materials and methods

### Experimental design

**Fig 1:**
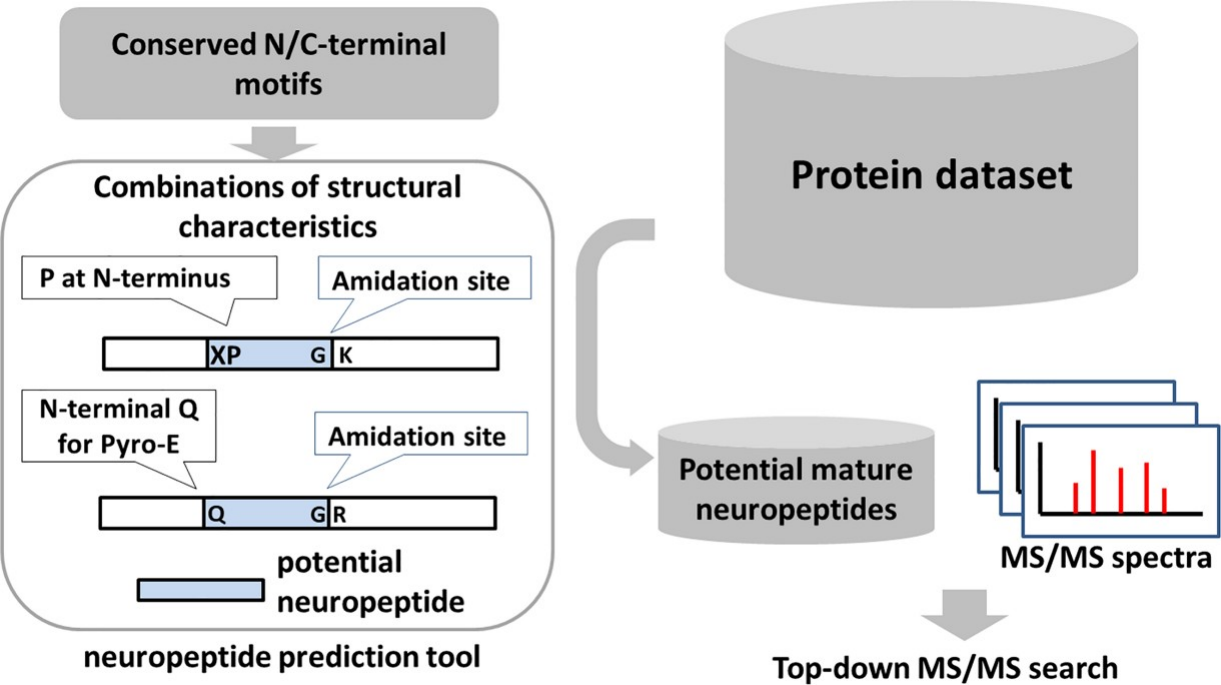
Schema of the neuropeptide identification strategy using a combination of peptide-spectrum matching against a dataset of *in silico* cleaved neuropeptide sequences extracted from putative neuropeptide precursors from the *Nematostella* protein database. Amino- and carboxyterminal motifs were used to scan the *Nematostella* protein database for neuropeptide precursor candidates, from which the peptides sequences were cleaved *in silico*. Peptide sequences were then exported into a target database for MS/MS spectral searching.

A comprehensive and highly sensitive neuropeptide identification pipeline was designed for *Nematostella vectensis*, using a combination of *in silico* neuropeptide prediction combined with tandem mass spectrometry (MS/MS). Fig 1 shows an overview of the approach used. First, the protein database of *Nematostella* was processed with a software tool to extract potential neuropeptide precursor sequences, based on their structural hallmarks. These include cnidarian-specific amino- and carboxyterminal cleavage motifs that flank the peptides sequences and their associated posttranslational modifications (PTMs). Fragmentation spectra were acquired by liquid chromatography-matrix-assisted laser desorption/ionization (LC-MALDI) MS/MS. Unlike conventional peptide-spectrum matching for naturally occurring peptides (as in peptidomics) or for enzymatic (tryptic) digests (as in proteomics), we narrowed the search space by using a smaller target dataset for peptide-spectrum matching. This smaller dataset only contains peptide sequences that are cleaved *in silico* from extracted putative peptide precursors from the *Nematostella* protein database. In contrast to searches in a conventional peptidomics workflow, which considers any amino acid residue as a potential cleavage site, the spectral matching search in the present method was performed directly against theoretical spectra of the mature forms of extracted neuropeptide sequences as in top-down proteomics.

### Sample preparation

Adult polyps of *Nematostella vectensis* originally collected from the Rhode River in Maryland [30] were kept in 1/3 artificial seawater at 18°C and fed three time per week with nauplius larvae of *Artemia salina*. The culture medium was changed once a week. Five adult polyps were homogenized with a probe sonicator in 1.5 mL methanol/water/formic acid (FA) solution (90:9:1) in 3 cycles (on for 5 s and off for 5 s) on ice. Large proteins were removed by centrifugation at 10,000 × *g* for 15 min, and the supernatant was transferred to new tube. The sample was freeze-dried using a vacuum centrifuge (Speedvac concentrator SVC200H, Savant, USA) and stored at -80°C for further treatment.

### MALDI-MS/MS mass spectrometry

The sample was pre-fractionated on a C18 column (BEH C18 column, Waters, Milford, MA, USA) using mobile phase at high pH (MilliQ and acetonitrile with ammonium hydroxide (20 mM), pH 10) and five fractions were made during the gradient (B: 5 to 90% in 30 min) at a flow rate of 100 μL /min. Pre-fractionated samples were further separated on a C18 column at low pH. The eluent was (A) water containing 0.5% FA and (B) 90% acetonitrile in 0.5% aqueous formic acid. The column was first washed and equilibrated with eluent A and 5% of eluent B. After loading the sample, a linear gradient from 5% B to 60% B in 60 min at a flow rate of 100 μL /min was used as the mobile phase. Thirty fractions of 200 μL were collected from the beginning of the gradient using an automatic fraction collector. The resulting samples were dried in a Speedvac and stored at -80°C until further analysis. Fractionated samples were resuspended in 1.5 μL water/ acetonitrile/FA (50/49.5/0.5 v/v). Subsequently, they were transferred onto a MALDI target plate (Bruker Daltonics, Bremen, Germany) and mixed with 1.5 mL of a saturated solution of CHCA in 50% acetonitrile containing 0.5% FA. After evaporation of the solvent, the MALDI target was introduced into the mass spectrometer ion source. Tandem mass spectrometry analysis was performed using the Ultraflex II instrument (Bruker Daltonics, Bremen, Germany) in a positive ion, reflectron mode. The instrument was calibrated externally with a commercial peptide mixture (peptide calibration standard, Bruker Daltonics). All spectra were obtained using Flex Control software (Bruker Daltonics, Bremen, Germany). The plate was initially examined in MS1 mode and spectra were recorded within a mass range from m/z 500 to 4000. Subsequently, the peaks with S/N 10 were selected and used for the optimized LIFT method from the same target. All tandem mass spectra were processed by means of the FlexAnalysis software (Bruker Daltonics, Bremen, Germany), and m/z values and intensities of each peak were recorded in peak list files.

### Mass spectrometry data analysis

An in-house software module was developed, enabling the prediction and extraction of potential neuropeptide sequences from *Nematostella* proteins as assembled from the genome at JGI (*Nematostella vectensis* version 1.0, all models) [31]. First, a protein was scanned for residues corresponding to the input C-terminal amino acid motif (e.g., GK or GR for C-terminal amidation). Second, an amino acid sequence corresponding to an N-terminal motif (e.g., XP or XXP; X is any amino acid)) was searched at the N-terminal side from the detected C-terminal motif within a given sequence length. In case multiple stretches matched N-terminal motif, they were considered potential N-termini of peptide sequences. All combinations of input amino acid motifs were applied. All peptide sequences that met the criteria were cleaved *in silico* and written in a FASTA formatted file that was subsequently used as a target database for peptide-spectrum matching.

To design the input motifs, we first assembled the sequences of all presently known cnidarian neuropeptides and their precursors from the UniProt sequence database and other public available resources (S1 Table) [23,25,32–39]. It is well known that bilaterian neuropeptide precursor proteins contain dibasic KR, KK, RK, and RR residues that flank the N- and C-termini of the peptides for cleavage of the peptides from their precursors [3]. We observed, however, that the already known cnidarian neuropeptide precursors rarely contain these dibasic residues (see S1 Table), which prompted us to search for cnidarian-specific input motifs. We observed that 52% of the cnidarian neuropeptide sequences had N-terminal prolines and 54% have N-terminal glutamines. In total, 84% had either an N-terminal proline or glutamine or both, indicating that these hallmarks can be used as input motifs to extract potential neuropeptide sequences from the *Nematostella* protein dataset. Currently known cnidarian neuropeptides also contain an amidation at their C-termini, which is a commonly observed PTM of neuropeptides across all animal species. Based on these observations, the following amino acid motifs were used to extract potential neuropeptide sequences from the *Nematostella* protein dataset:

C-terminal motif:

GK↓ or GR↓: glycine, required for amidation, before K or R

N-terminal motif:

↓XP: proline from the second position from N-terminus

↓XXP: proline from the third position from N-terminus

↓Q: glutamate for the formation of pyroglutamic acid

(↓ indicates the cleavage site)

C-terminal K or R of the extracted peptides were then removed and the saved sequences were stored as a FASTA formatted file.

For comparison, another set of potential neuropeptide sequences was created in a similar way, but now making use of the processing sites commonly found in bilaterian neuropeptide precursors, such as those for prohormone convertases (PC1-3) and for furin [40]. These include the dibasic amino acid sites (KK, RR, KR, RK), as well as sites containing monobasic amino acid residues separated by 2, 3, or 6 other residues (e.g. RXXR, KXXK) [2]. Extraction of potential neuropeptide sequences was thus achieved by applying the following cleavage pattern according to Falth *et al*.: **(K/R) Xm(K/R)** ↓X_k_**(K/R) Xn(K/R)** ↓. where m and n = 0, 2, 4, 6; X can be any amino acid residue and k = 6–40 [41]. Residues in bold were subsequently removed and the sequence X_k_ was stored and saved as FASTA formatted file. To create decoy sequences, the amino acid sequences of the original protein dataset were randomly rearranged and subjected to the aforementioned peptide prediction and extraction processes.

The FASTA files used in the present study is available online.

The software was coded in C++ using Visual Studio 2008 (Microsoft) and Boost library. It is available online (https://sourceforge.net/projects/enpg/) with the source code under the MIT license.

The obtained tandem mass spectra were searched against three datasets for comparison: (i) a database holding all *Nematostella* proteins (ii) a database containing predicted neuropeptide sequences by applying cnidarian neuropeptide precursor hallmarks and (iii) a database containing predicted neuropeptide sequence by applying common bilaterian neuropeptide precursor processing motifs (**(K/R) Xm(K/R)** ↓X_k_**(K/R) Xn(K/R)** ↓). The Mascot search engine version 2.3 (Matrix science, London, UK) was used. Peptide-spectrum matching was performed, allowing C-terminal amidation of glycine extended peptides, pyroglutamic acid (Q and E), and oxidation (M), all being common posttranslational modifications of neuropeptides. Searches against the reduced dataset containing the predicted peptide sequences were performed with “No Cleavage” setting, in which cleavage of input sequences was not considered. Precursor masses were matched to the theoretical masses of intact peptide sequences. Mass tolerance for precursor and fragment ions were set to 0.4 and 0.8 Da. Peptides were first tentatively identified with a Mascot expect value less than 0.05; then, they were further confirmed by manual verification of the product ions assigned. Decoy search was conducted by using the aforementioned decoy FASTA file. The presence of an N-terminal signal peptide in the protein precursor of a mass spectrometry-identified peptide was examined using SignalP (version 5) [42]. The output files of SignalP can be found in the supporting materials.

### Expression analysis

Partial ORF sequences for the identified peptides were obtained from the National Center for Biotechnology Information (NCBI) trace archive of *Nematostella vectensis* (data generated by the Joint Genome Institute) and from *Stellabase* (http://cnidarians.bu.edu/stellabase/index.cgi). Gene-specific primers were designed based on the ORF sequences. The primer sequences are available in S3 Table. PCR products for the neuropeptide-encoding genes were subcloned into pGEM-T (Promega) and sequenced. Riboprobes were synthesized and purified as described previously [43]. *In situ* hybridization was performed as previously described [44], with the following modifications: specimens were fixed with 4% paraformaldehyde/PBS + 0.1% Tween 20 (PBST) for 1 h, washed with methanol 3 times and stored at -20°C. Hybridization of 0.4- to 1.1-kb digoxygenin (DIG)-labeled antisense RNA probes was carried out using hybridization solution containing 1% SDS at 50–65°C for at least 22 h. For post-hybridization washes, specimens were washed by serial dilutions (75%, 50%, and 25%) of hybridization solution with 2× SSC at 55°C. After DIG-labeled probe was visualized using BM purple (Roche), specimens were washed with PBST.

## Results

### Peptide identification by MALDI MS/MS

**Fig 2:**
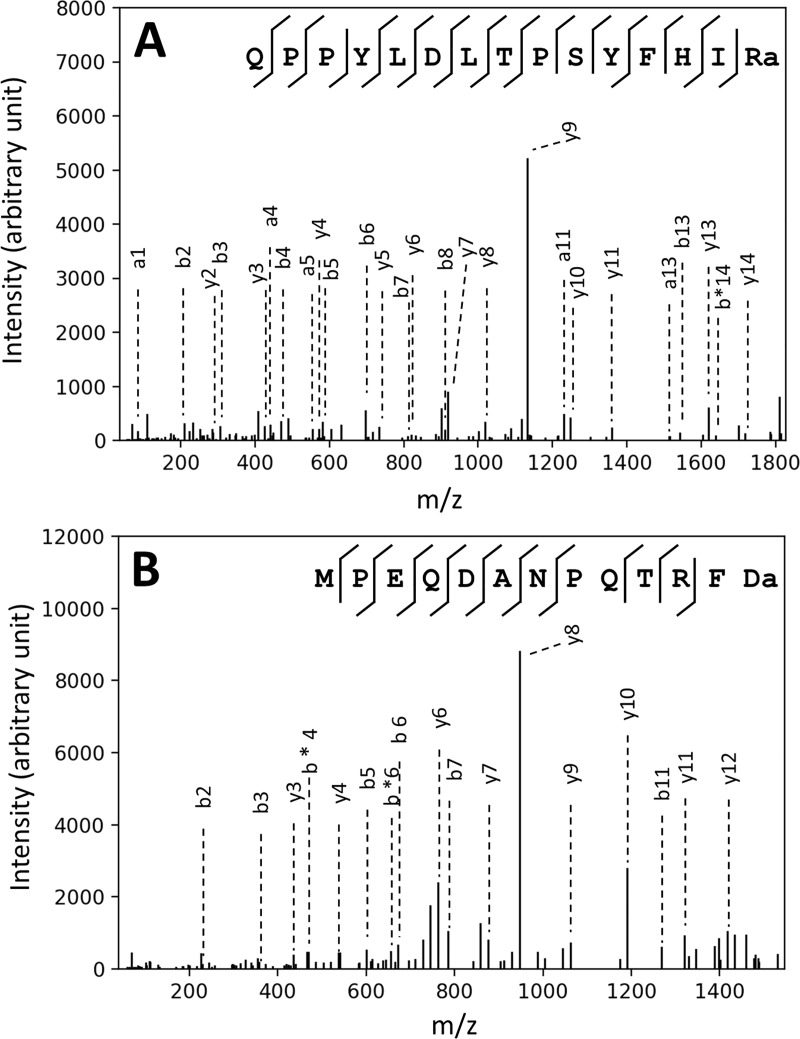
Representative fragment spectra of identified peptides. Fragmentation spectra of the peptide “QPPYLDLTPSYFHIRa” (A) and “MPEQDANPQTRFDa” (B). The dotted lines indicate fragment ions assigned. Ion labeled with * means loss of NH3.

**Fig 3:**
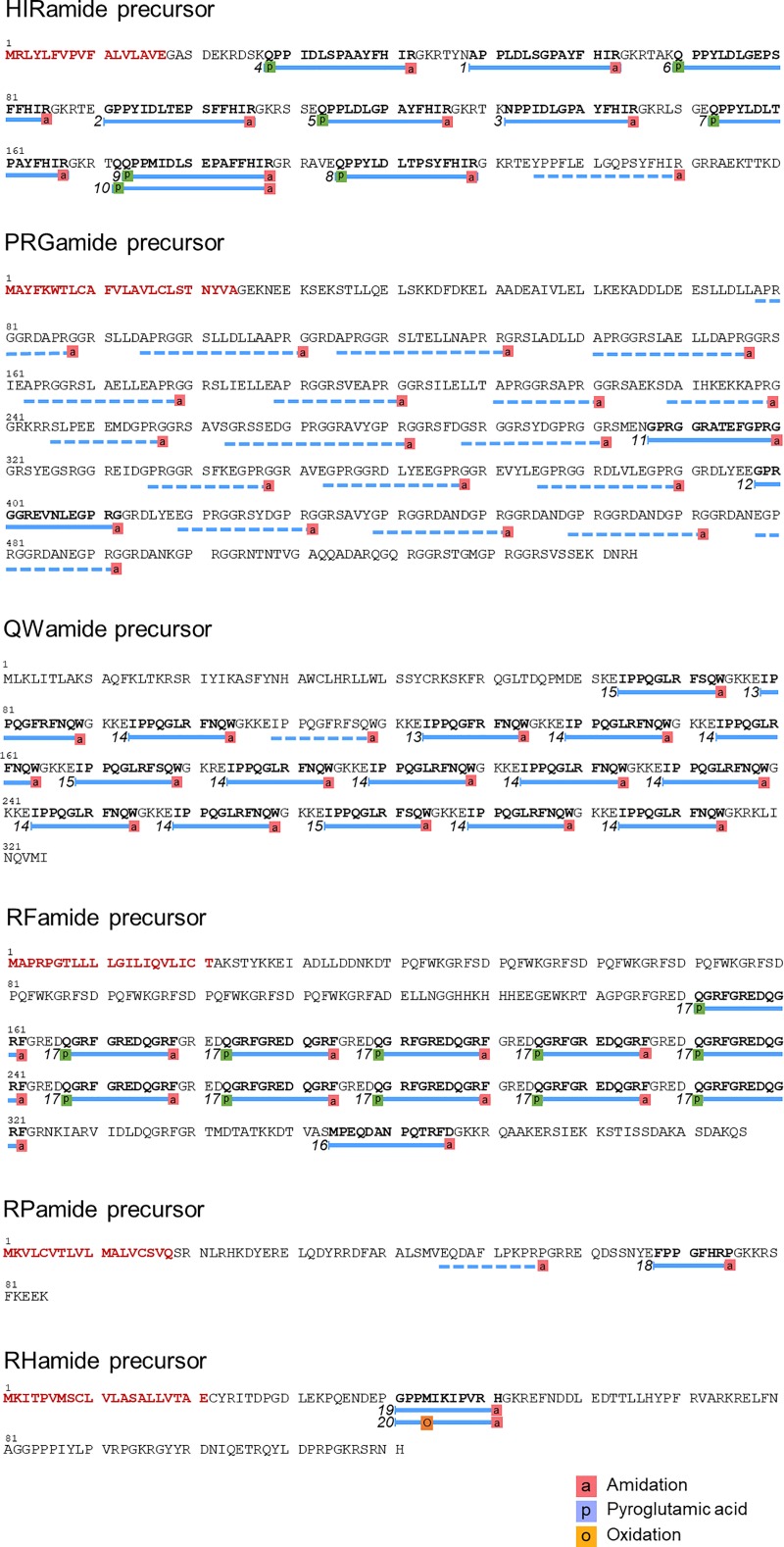
Primary structures of neuropeptide precursor proteins. The location of the detected neuropeptides is indicated by full lines. Numbers correspond to the ID in Table 1. Predicted neuropeptides that were not detected in this study are indicated by dotted lines. Signal peptides predicted by SignalP are highlighted in red.

The tandem mass spectra were acquired from off-line LC-MALDI MS/MS analysis of a peptide extract of *Nematostella vectensis*. Peptide identification was carried out using the Mascot peptide-spectrum matching tool, considering pyroglutamic acid formation, C-terminal amidation of glycine extended peptides, and methionine oxidation. In order to reduce the search space for peptide spectrum matching, we employed a reduced dataset that only comprised potential neuropeptide sequences that were extracted and cleaved from the *Nematostella* protein dataset using cnidarian-specific amino acid motifs as input queries *in silico*. This way, 400 to 800 peptides were extracted, efficiently reducing the size of the search space. This dataset was then used as a target sequence dataset for peptide-spectrum matching, and the search was done in the top-down fashion with “no-cleavage” setting. Twenty unique peptides were identified with a Mascot E-value < 0.05. Table 1 shows the identified peptide sequences. The representative fragment spectra used for neuropeptide identifications are shown in Fig 2. The amino acid sequences and the details of their precursor protein coding genes can be found in Fig 3 and S2 Table.

**Table 1:** Sequences of detected peptides and their Mascot E-values.

				Mascot E-value	
Peptide name	ID	Peptide sequence	Peptide database Cnidarian-specific motifs	Protein database No motif	Peptide database- bilaterian neuropeptide processing motif
HIRamide	1	APPLDLSGPAYFHIRa	**1.9E-02**	4.2E+00	nd
	2	GPPYIDLTEPSFFHIRa	**8.8E-09**	**2.1E-06**	nd
	3	NPPIDLGPAYFHIRa	**7.3E-07**	**1.6E-04**	**5.9E-07**
	4	pQPPIDLSPAAYFHIRa	**1.1E-04**	**2.5E-02**	**7.7E-05**
	5	pQPPLDLGPAYFHIRa	**5.0E-07**	**1.1E-04**	nd
	6	pQPPYLDLGEPSFFHIRa	**4.5E-04**	1.0E-01	**3.6E-04**
	7	pQPPYLDLTPAYFHIRa	**2.5E-04**	5.5E-02	nd
	8	pQPPYLDLTPSYFHIRa	**5.4E-06**	**1.2E-03**	nd
	9	pQPPMIDLSEPAFFHIRa	**1.2E-06**	**2.7E-04**	nd
	10	pQQPPMIDLSEPAFFHIRa	**1.7E-03**	3.5E-01	nd
PRGamide	11	GPRGGRATEFGPRGa	**1.2E-04**	**2.8E-02**	nd
	12	GPRGGREVNLEGPRGa	**6.7E-03**	1.7E+00	nd
QWamide	13	IPPQGFRFNQWa	**3.4E-02**	8.2E+00	nd
	14	IPPQGLRFNQWa	**2.9E-03**	6.7E-01	nd
	15	IPPQGLRFSQWa	**2.8E-03**	6.4E-01	nd
RFamide	16	MPEQDANPQTRFDa	**2.3E-05**	**5.3E-03**	nd
	17	pQGRFGREDQGRFa	**3.2E-03**	6.3E-01	nd
RPamide	18	FPPGFHRPa	**2.0E-03**	4.9E-01	nd
RHamide	19	GPPMIKIPVRHa	**2.0E-03**	5.0E-01	nd
	20	GPPMoIKIPVRHa	**7.7E-04**	2.5E+01	nd

E-values lower than the threshold (0.05) are indicated in bold. Mascot E-values in the first column result from peptide spectrum matching (PSM) against the smaller dataset of peptides extracted *in silico*. Mascot E-values in the second column result from PSM against the *Nematostella* protein database. Mascot e-values in the third column result from PSM against a database of peptide sequences that were extracted from the *Nematostella* protein database using the most common neuropeptide processing motif, which is based on the presence of dibasic cleavage sites as substrates for prohormone convertases and carboxypeptidase E in bilaterian neuropeptide precursors. C-terminal amidation, oxidation, and N-terminal pyroglutamic acid are indicated as “a”, “o,” and “p,” respectively. PC: protein convertase; nd: not detected.

We compared the performance of our peptide identification approach, using peptide-spectrum matching against a reduced cnidarian-dedicated dataset, with the conventional peptidomics approach that instead uses the entire protein dataset.

As shown in Table 1, the e-values in the spectral search against the reduced dataset of predicted *Nematostella* peptides (first column) significantly improved compared to those of the search against the entire *Nematostella* protein dataset (second column). Second, spectral searching against the reduced dataset yielded more than twice as many peptide identifications. Indeed, the E-values of the peptide hits in the second column (search against entire *Nematostella* protein dataset) were not only distributed in the higher range; many of them were also similar to the E-values of the decoy search result (S1 Fig), which evidently complicates the peptide identification process. In contrast, the E-values of the peptide hits in the first column (search against the reduced dataset of predicted candidate *Nematostella* peptide sequences) were distributed in a lower range, showing clear separation from the range of E-values of the decoy search result (S1 Fig).

By comparison, scanning the much larger search space of the entire *Nematostella* protein dataset, without any cleavage site specification (which is the conventional peptidomics search), revealed about 100,000 to 200,000 peptide sequences that matched the molecular masses of the mass spectrometry-detected peptides in the sample. In this third peptide spectrum matching search, we therefore used the most common cleavage rule of neuropeptide precursors, instead of the ‘no cleavage setting’. This includes sites containing 2 basic amino acid residues (KK, RR, KR, RK), as well as sites containing pairs of basic amino acid residues separated by 2, 3, or 6 other residues (e.g. RXXR, KXXK), followed by removal of C-terminal basic residues [2]. In this approach proposed by Falth *et al*, the search space is also narrowed, preserving only potential neuropeptide structures that are cleaved through the most common cleavage patterns in bilaterian neuropeptide precursors. [41]. However, the third column in Table 1 shows that only 3 peptides could be identified this way. This poor peptide identification indicates that bilaterian-typical dibasic prohormone convertase cleavage sites are not commonly utilized for neuropeptide precursor processing in *Nematostella*, which is in accordance with neuropeptide precursor sequences identified in other cnidarian species [33,36,45–47].

### Novel neuropeptides in *Nematostella vectensis*

Most of the peptides identified in the present study have never been reported so far. Their protein-coding genes have not been annotated as neuropeptide precursor genes, except for the RFamide precursor. Fig 3 shows the structures of the newly identified peptide precursors containing the mass spectrometry-identified peptides. All precursors start with a signal peptide at their N-terminus, typical for proteins destined for secretion, except for the QWamide precursor, which, however, contains several hydrophobic amino acid residues at the N-terminus. Overall, the identified peptide precursors contain multiple copies of the mature peptide sequences sharing the same C-terminal motifs. It should be noted that the precursors also contain multiple other potential neuropeptide sequences that display the same C-terminal motif as the identified peptides, as well as the structural motifs that we used for the *in silico* peptide extraction. For instance, the precursor protein of HIRamide peptides contains an additional putative peptide that shows the same HIRamide motif at C-terminus, together with the amidation motif and a proline at the second and third position from the N-terminus. It remains elusive why these peptides were not detected in our analysis. Either these peptides are not produced, or their presence was below the detection limit of the mass spectrometer.

**Fig 4:**
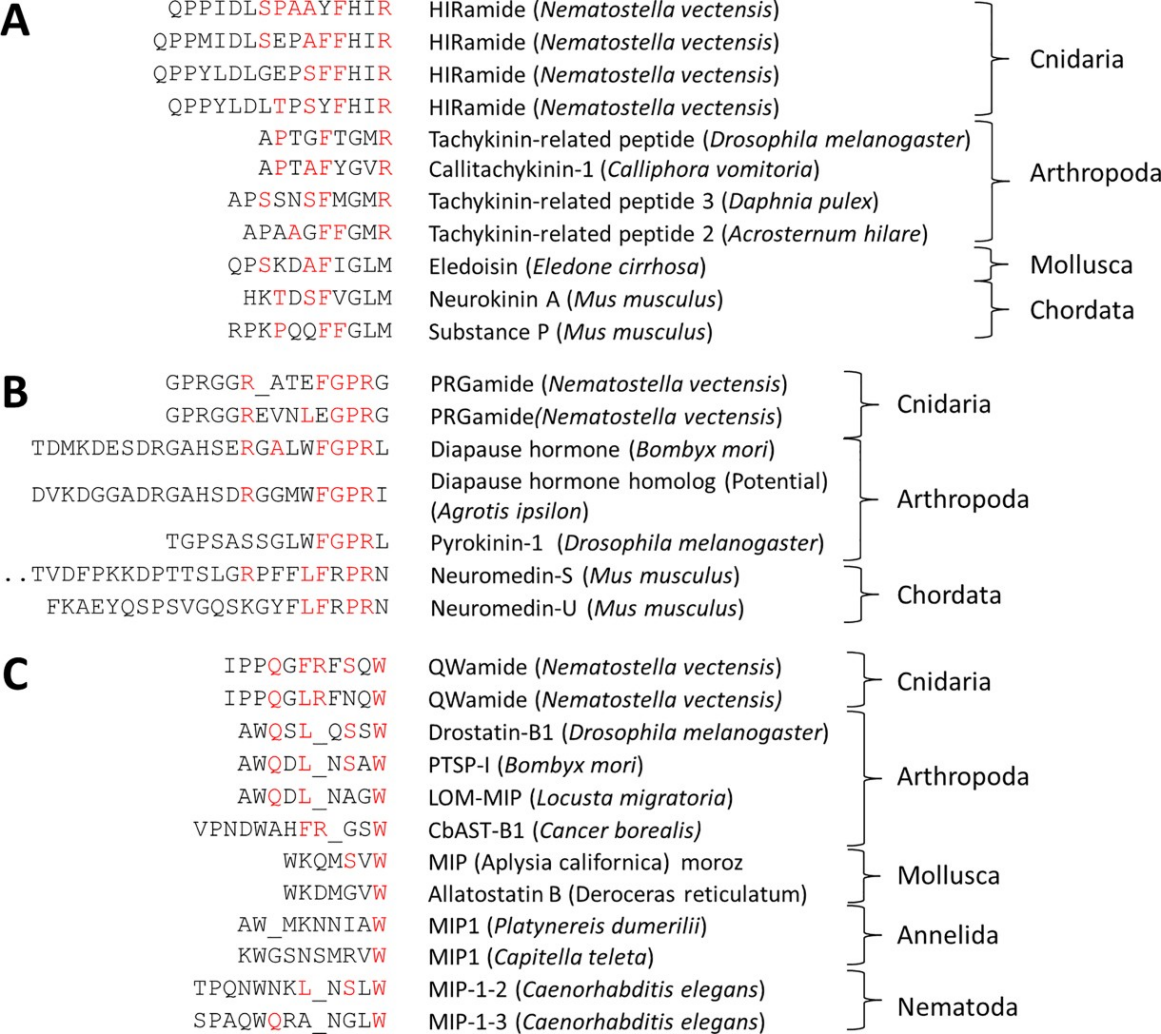
Structural similarities of identified neuropeptides in *Nematostella vectensis* and other species. A: HIRamides and Tachykinin related peptides[18,48–52]. B: PRGamides and PRXamide related peptides [53–57]. C: QWamides, myoinhibitory peptide (MIP) and allatostatin type B [58–64]. Conserved amino acid residues are shown in red.

The HIRamide peptide precursor contains 9 structurally related peptide sequences and a typical signal peptide sequence at the N-terminus. The HIRamide peptides share sequence similarities with arthropod tachykinin peptides at their C-termini (Fig 4A). Tachykinins and related peptides are well studied neuropeptides that have been evolutionarily conserved in both protostome (nematodes, arthropods, annelids, and mollusks) and deuterostome animals (echinodermates and chordates). Based on their C-termini, tachykinin and related peptides can be further classified into two subfamilies: the “-FXGLMa” and “-GFXGXRa” subfamilies. *Nematostella* HIR peptides display more similarities to the “GFXGXRa” subfamily, as indicated by the aromatic phenylalanine or tyrosine residues at the fifth position from the C-terminus and by the C-terminal arginine residue. Arthropod tachykinins and Nematostella HIRamides share the PXXFYXXRamide motif.

The RFamide neuropeptide family is widespread among bilaterian organisms and RFamides have previously been reported in cnidarians including the sea anemone. In this study, an additional member of the RFamide family, MPEQDANPQTRFDa, was identified within the RFamide precursor protein [29]. This peptide has an additional asparagine residue at the C-terminus, in contrast to other peptides contained in this precursor, which all display the carboxyterminal RFamide motif.

Two peptides, GPRGGRATEFGPRGamide and GPRGGREVNLEGPRG, share the C-terminal motif GPRGamide. The corresponding peptide precursor contains many peptide copies, which all share this motif. These cnidarian peptides seem to display sequence similarities with protostomian pyrokinins that are characterized by the C-terminal FXPRLamide sequence (Fig 4B) [65,66].

Three peptides derived from a single precursor protein display either the C-terminal sequence motif FSQWamide or FNQWamide (Fig 4C). This motif aligns with the motif that typifies bilaterian myoinhibitory peptide/allatostatin type B peptide family, which is a neuropeptide family widespread among protostomes with various functions such as inhibitory effect on muscles, juvenile hormone synthesis, mating behavior, sleep and learning. [60,67–70].

In addition to the peptides described above, we discovered two groups of peptides (RPamide and RHamide) that originated from two precursor proteins. So far, we have not found any sequence similarities to known neuropeptide families (Table 1).

### Expression patterns of HIRamide, PRGamide, and VRHamide neuropeptides

**Fig 5:**
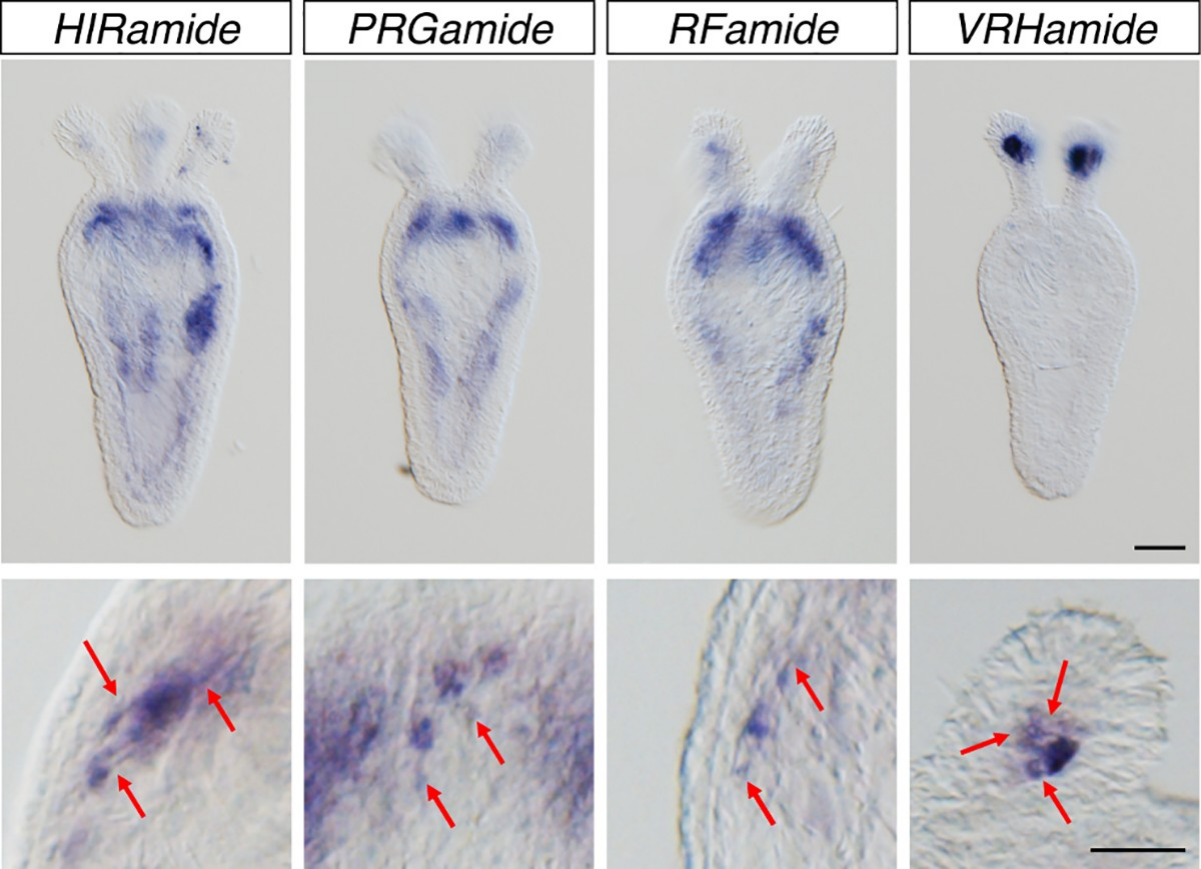
WISH staining of juvenile polyps of *Nematostella vectensis* (8 days post fertilization). The figure shows localized expression of *HIRamide, PRGamide RFamide*, and *VRHamide* genes at low (upper panels) and high magnification (lower panels). Scale bars, 100 μm (upper) and 50 μm (lower). Neural processes are indicated by red arrows.

Our whole mounts in situ hybridization (WISH) analyses confirmed that the peptide genes encoding the identified peptides HIRamide, PRGamide, and VRHamide are exclusively expressed in neurons. As shown in Fig 5, the expression of all peptide genes as detected by WISH was observed in specific cells housed mainly in the endodermal layer at the juvenile polyp stages. Careful observation of the morphology of the positively stained cells showed round-shaped cell bodies with neurite-like processes (Fig 5, lower panel). This indicates that the genes encoding the detected peptides are expressed in neurons, and that the newly identified peptides are true neuropeptides. The genes encoding HIRamide and PRGamide neuropeptides were strongly expressed in neuronal subsets around the mouth opening (Fig 5A), indicating that these neurons develop at the oral side to form region-specific neural network. The tissue around the mouth of cnidarian polyps has been shown to express a number of neuronal markers including RFamides, which are evolutionarily conserved in metazoans (Fig 5, upper panel) [28], and to develop a regionalized nervous system, which is known as the oral nervous system (*Nematostella*) or the nerve ring (*Hydrozoa*) [71–73]. The expression pattern of the VRHamide-encoding gene was in sharp contrast with that of HIRamide, PRGamide, and RFamide-encoding genes. Expression of this gene could not be detected at the oral tissue. Instead, VRHamides were strongly and exclusively detected in neurons located at the most distal region of the tentacle endoderm (Fig 5, upper panel). This unexpected and interesting expression pattern suggests a specific function of VRHamides in development and/or in contractility of the tentacles.

## Discussion

We studied the neuropeptidome of *Nematostella vectensis*, a sea anemone that belongs to the Cnidaria phylum, possessing one of most basal nervous systems in the Animal Kingdom. Recent advances in mass spectrometry technologies have greatly enhanced the identification of neuropeptidomes from biological matrices [14,74–76]. However, an effective bioinformatics solution for neuropeptide identification is still missing and leaves the interpretation of tandem mass spectral data difficult. We overcame this difficulty by performing peptide spectrum matching against a narrowed database comprising *in silico* cleaved putative neuropeptide sequences. We successfully identified 20 peptides encoded in six neuropeptide precursor genes from *Nematostella* and experimentally confirmed that at least four precursor genes are expressed in neurons exhibiting distinct neurophysiological activities. Our approach can be easily incorporated with recent advanced technologies in peptidomics, and such a combination will greatly improve the analysis of the neuropeptidome in a broad range of biological samples.

### Strategies for the improvement of peptide identification

Peptide-spectrum matching has become the main tool for identifying naturally occurring peptides. Unlike classical methods, such as Edman degradation, this approach takes advantage of the high sensitivity of modern mass spectrometry instruments and high throughput peptide search engines, therefore enabling comprehensive neuropeptide characterization. One of the main difficulties in peptidomics is that the peptide-spectrum matching tool needs to used with the “no enzyme” setting, which results in an enormous search space. Consequently, the peptide-spectrum matching result is usually worse in comparison with a peptide search in a proteomics setting.

The peptide characterization strategy employed in this study is a combination of predicting potential neuropeptide sequences and peptide-spectrum matching. This approach is effective in narrowing the search space and thereby significantly increases the sensitivity of peptide identification. Fälth *et al*. employed a similar approach to narrow search space in order to improve peptide-spectrum matching [41]. They used the most common processing rule of neuropeptide precursors to extract potential neuropeptide sequences from a large protein database, which significantly improved peptide identification rate. We tested their approach by extracting peptide sequences from the *Nematostella* protein dataset using the most common cleavage sites containing basic residues, but failed to identify the majority of *Nematostella* neuropeptides identified by the present strategy. This is explained by the fact that the most common basic amino acid cleavage site in bilaterian neuropeptide precursors seems to be rare in cnidarian (*Nematostella*) neuropeptide precursors.

Southey *et al*. developed a dedicated tool to predict basic cleavage sites in neuropeptide precursor genes [77]. The prediction engine, NEUROPRED, needs to be trained with the information of known neuropeptides and their precursors; hence many identified peptides and information of processing sites are required. Compared to the prediction with simple motif matching, the prediction by NEUROPRED using logistic regression modeling is more reliable, but, on the other hand, rather strict and potentially discards many forms of cleaved peptides, especially if the large amount of training data is not available. These approaches are very effective for the prediction of neuropeptides in bilaterian phyla where a lot of sequence information is already available. In contrast, the number of known cnidarian neuropeptides is limited compared to the massive amounts of peptide sequences in bilaterian phyla. Cnidarian neuropeptides display, however, very clear N/C-terminal motifs. The limited number of peptide sequences and highly conserved motifs in cnidarian neuropeptides make regression modeling unnecessary and less effective for peptide identification in this animal group.

Unlike bilaterian animals, non-basic cleavage sites are commonly observed in cnidarian neuropeptide precursors [33,34,37,39,45–47,78]. In fact, most of the peptides identified in the present study were generated via cleavages at non-basic residues. Therefore, the approach we employed in the present study (i.e., the prediction of putative neuropeptide sequences based on other structural hallmarks, such as the N-terminal pyroglutamation or Proline at the second or third position from the N-terminus and the C-terminal amidation) is more comprehensive and effective to preserve as many potential neuropeptide sequences as possible in the target database for peptide-spectrum matching. Nevertheless, the approach also has a drawback in that it only allows identification of neuropeptides that display these specific features. Non-amidated peptides or peptides that are not N-terminally protected are not contained in this reduced database.

Noteworthy, prohormone convertases distinct from the furin/subtilisin family likely exist in other bilaterians as has been suggested in several peptidomic and non-peptidomic studies. Hence, our method may eventually reveal novel peptides in bilaterian species [13,79–83].

### Neuropeptide evolution

Many pioneer studies in the late 80s and early 90s already pointed to homologies between protostomian and deuterostomian neuropeptides based on neuropeptide sequence identifications in vertebrate, insect, molluscan, and cnidarian species [20,36,84–88]. However, until today, knowledge on signaling molecules, including neuropeptides, in the more ancient eumetazoan animals remained scarce. A previous study showed a highly conserved set of genes in the *Nematostella vectensis* genome, including molecules involved in neurotransmission [28,89]. Anctil *et al*. has reported potential neuropeptide coding genes by means of homology searching [29]. Although these predicted precursor proteins contain short repeats of neuropeptide-like motifs, it is difficult to prove the peptide identities as the corresponding precursors were predicted solely based on their protein sequence without any experimental evidence that the peptides are processed. Some of the neuropeptide precursor proteins predicted by Anctil showed a significant degree of structural similarities to non-peptide precursors, leaving doubts on their identity as true neuropeptide precursors. In the present study, we were unable to identify any of those predicted peptides, neither by conventional “no-enzyme” searches against the entire *Nematostella* protein database, nor by searches against the reduced dataset of predicted peptides. Homology-based searches based on short neuropeptide sequences remain a difficult task. More importantly, the homology-based prediction of neuropeptide precursor genes does not provide evidence for the occurrence of the predicted neuropeptides *in vivo*. Therefore, peptide identification approaches that are based on conventional biochemical purification methods and powered by upcoming bioinformatic technologies are necessary to empirically prove the predicted peptides. They will be important to identify not only evolutionary conserved peptides, but also evolutionarily derived or species-specific peptides that correspond to the diverged physiological traits.

Our data have also important implications on our insight into the evolution of neuropeptides. Since the neuropeptide repertoire in ancient eumetazoans is largely unknown, the origin of most of neuropeptides found in bilaterian animals remains difficult to reveal. Jekely (2013) performed a similarity-based clustering analysis of genes encoding neuropeptides and neuropeptide GPCRs across metazoan phyla and concluded that the last common ancestor of eumetazoans had various small amidated peptides including RFamide, RYamide, and Wamide) [90]. Ancestral bilaterian neuropeptide-receptor families include GnRH, vasopressin, GnIH/SIFamide, CRF/diuretic hormone, calcitonin/DH31, NPY/NPF, neuromedin-U/pyrokinin, CCK/sulfakinin, galanin/allatostatin-A, and orexin/allatotropin [90,91]. It has been suggested that these neuropeptide families may have originated concomitantly with the origin of a complex bilaterian body plan, with control of food intake and digestion, excretory and circulatory systems, light-controlled reproduction, a centralized nervous system, complex reproductive behavior, and learning.

Thus far, the urbilaterian origin of neuropeptide signaling pathways remains largely elusive. Nevertheless, the present study strongly suggests that the extent of the conservation of some neuropeptide families may even be deeper than previously proposed. Indeed, we were able to identify neuropeptides structurally related to the myoinhibitory peptide/allatostatin type B, tachykinin, and neuromedin-U/pyrokinin families in the cnidarian species, *Nematostella*, suggesting that these neuropeptide families were already present in the common ancestors of all eumetazoan species. Neuropeptides modulate various biological processes by signaling through G protein coupled receptors (GPCRs). The *Nematostella* genome contains at least 79 GPCR coding genes structurally related to known neuropeptide receptors in bilaterians [29]. However, at this moment, in any of the four non-bilaterian phyla–Porifera, Placozoa, Ctenophora and Cnidaria–functional studies showing which neuropeptides signal through which GPCRs are still lacking, but at least they are now on the horizon for cnidarians.

Taken together, there are still many missing pieces in the ‘jigsaw puzzle’ of neuropeptide evolution, but we anticipate that the discovery of the receptors, downstream targets, and functions of cnidarian representatives of ancient eumetazoan neuropeptide families will provide important new insights into the evolution of neuropeptide functions in the Animal Kingdom.

## Supporting information

S1 File Output files of SignalP. Original output files of SignalP analysis on identified neuropeptide precursor proteins.
(ZIP)

S1 Fig The distribution of e-values of PSMs. Each circle indicates the e-value of the top-scoring hit in the dataset. A: Search against the predicted neuropeptide dataset. B: decoy of the predicted neuropeptides dataset. C: the whole protein models. D: decoy of the whole protein models.
(DOCX)

S1 Table The structures of the previously identified cnidarian amidated neuropeptides. (TSV)

S2 Table Details of the neuropeptide precursor genes. (XLSX)

S3 Table Primer and cDNA sequences of neuropeptides. (XLSX)
