# Supplementary material for: A combined strategy of neuropeptide prediction and tandem mass spectrometry identifies evolutionarily conserved ancient neuropeptides in the sea anemone Nematostella vectensis
Source: PLoS One. 2019 Sep 23;14(9):e0215185. doi: 10.1371/journal.pone.0215185 (PMC6756747; doi:10.1371/journal.pone.0215185)
Supplement: S1 Fig — Each circle indicates the e-value of the top-scoring hit in the dataset. A: Search against the predicted neuropeptide dataset. B: decoy of the predicted neuropeptides dataset. C: the whole protein models. D: decoy of the whole protein models. (DOCX) [file pone.0215185.s002.docx]

**
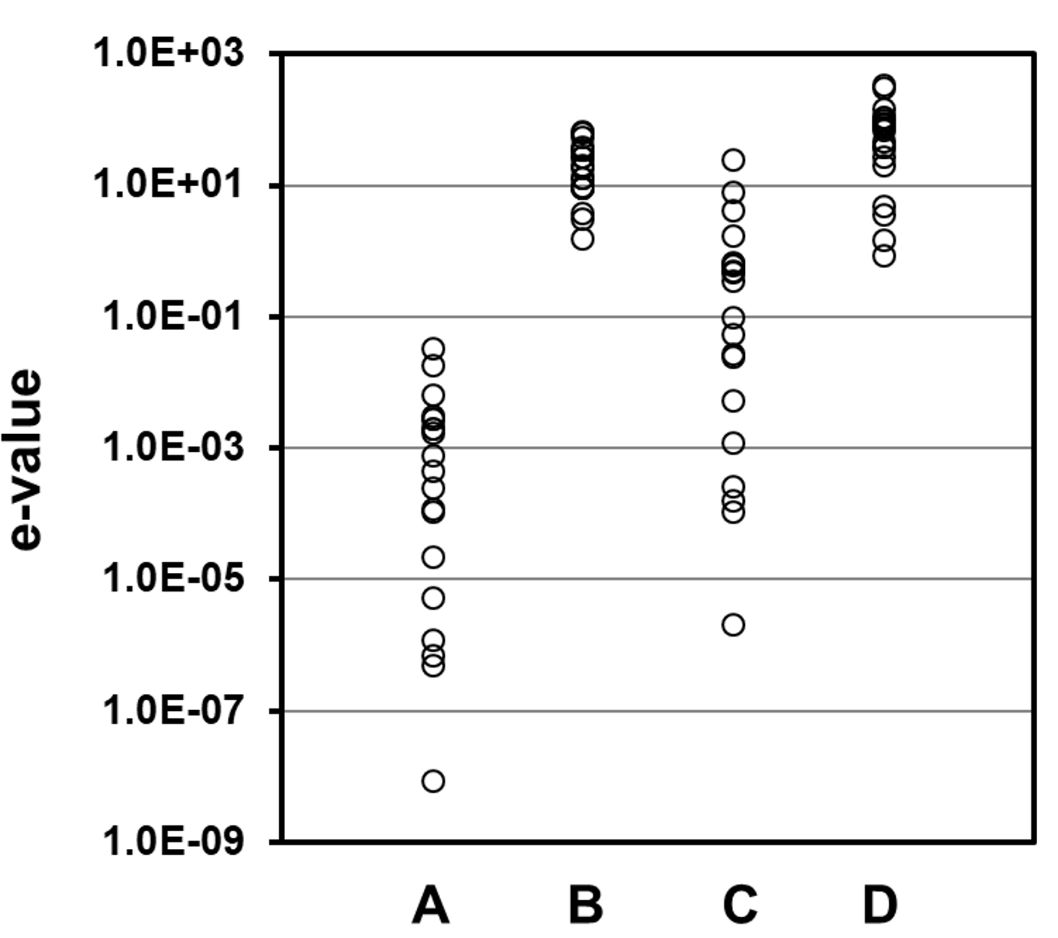
**

**Fig 2.** **The distribution of e-values of the hits found in the datasets.** Each circle indicates the e-value of the top-scoring hit in the dataset. A: Search against the predicted neuropeptide dataset. B: decoy of the predicted neuropeptides dataset. C: the whole protein models. D: decoy of the whole protein models.
